# Contagious yawning is stronger in wolves than in dog-admixed wolves

**DOI:** 10.1007/s10071-026-02086-w

**Published:** 2026-08-19

**Authors:** Federica Amici, Katja Liebal, Linda Oña, Mattia Iacuzzi, Tina Altdörfer, Arne Gretschzel, George Kamanga, Manon Delaunay, Paolo Ciucci

**Affiliations:** 1 Life Sciences, Institute for Biology, Human Biology and Primate Cognition, Leipzig University, Leipzig, Germany; 2 Max Planck Institute for Evolutionary Anthropology, Department of Comparative Cultural Psychology, Leipzig, Germany; 3 Department of Biology and Biotechnology ‘‘Charles Darwin”, Sapienza University of Rome, Rome, Italy

## Abstract

Behavioural contagion occurs when the behaviour of one individual elicits the same behaviour in another individual observing the event, and it is considered one of the fundamental mechanisms promoting group coordination and cohesion in social species, including humans. Empirical research on behavioural contagion has focused primarily on yawning and has shown evidence of it in several animal species. Here, we studied behavioural contagion in 2 groups of captive wolves (N = 20) and 3 groups of captive dog-admixed wolves (N = 13). We conducted behavioural observations of naturally occurring yawning events and monitored the yawning behaviour of the other group members in the two minutes following each event. Our results showed that, in both admixed and non-admixed wolves, individuals who had the opportunity to observe the initial yawn were significantly more likely to yawn within the subsequent two minutes than those who had not observed the event. Crucially, this effect was stronger in non-admixed than in admixed wolves. Furthermore, individuals that were more socially integrated were more likely to elicit behavioural contagion in both admixed and non-admixed wolves. In contrast, neither individual rank nor dyadic relationship quality between the yawner and the observer mediated the probability of contagion. By revealing variation in behavioural contagion patterns between admixed and non-admixed wolves, this study provides insights into how introgressive hybridization with domestic species may influence mechanisms underlying social coordination and pack cohesion in wolves.

**Supplementary Information:**

The online version contains supplementary material available at 10.1007/s10071-026-02086-w.

## Introduction

The ability to coordinate behaviour with conspecifics can confer substantial fitness benefits to group-living animals, for example by strengthening social cohesion, facilitating collective vigilance, improving the effectiveness of anti-predator strategies and increasing foraging efficiency through cooperative hunting (Conradt and Roper 2000; Duranton and Gaunet 2016; Gallup and Gallup 2007; Lakin et al. 2003). One mechanism that may support such coordination is behavioural contagion, which occurs when observing the behaviour of one individual increases the probability of performing the same behaviour (Zentall 2003; Palagi et al. 2020). By promoting temporal alignment of activities within dyads and groups, behavioural contagion may contribute to behavioural synchrony without requiring explicit signalling or complex cognitive skills, and it is therefore considered a powerful tool to facilitate coordination and collective behaviour (Duranton and Gaunet 2016; Massen and Gallup 2017).

Behavioural contagion is thought to arise from simple perception–action mechanisms such as non-conscious mimicry, whereby observing a behaviour automatically activates the corresponding motor representation in the observer, without requiring explicit representation of others’ internal states (Chartrand and Bargh 1999; Lakin et al. 2003; Yoon and Tennie 2010). Empirical research on behavioural contagion has focused primarily on yawning, and to a lesser extent on other behaviours such as scratching, laughter or play (Massen and Gallup 2017). The primary focus on yawning likely stems from its being relatively common, highly stereotyped and easily identifiable (Provine 1986; Yoon and Tennie 2010), which makes it particularly suitable also for observational settings. Spontaneous yawning is widespread in vertebrates and has been associated to a variety of physiological functions, including arousal regulation, thermoregulation and brain oxygenation (Baenninger 1997; Gallup 2010; Massen et al. 2014; Smith 1999). In contrast, contagious yawning is triggered by social stimuli and appears to occur in fewer taxa than spontaneous yawning (Massen and Gallup 2017). Supporting this notion, experimental studies have failed to show contagious yawning in some species that are solitary or facultatively social, such as red-footed tortoises (Wilkinson et al. 2011). This absence suggests that contagious yawning is unlikely to arise solely from a simple perceptual-motor reflex and may instead depend on the underlying social or cognitive conditions.

Evidence for contagious yawning has been reported primarily in non-human primates, including most great apes (e.g., Amici et al. 2013; Anderson et al. 2004; Campbell et al. 2009; Demuru and Palagi 2012; Palagi et al. 2014), several catarrhines (e.g., Gallo et al. 2021; Palagi et al. 2009; Paukner and Anderson 2006) and, more recently, both platyrrhines (Valdivieso-Cortadella et al. 2023) and strepsirrhines (Lemes et al. 2024; Valente et al. 2023). Beyond primates, evidence for contagious yawning has also been reported in other taxa, including wolves (*Canis lupus*; Romero et al. 2014), pigs (*Sus scrofa*; Norscia et al. 2021), sheep (*Ovis aries*, Yonezawa et al. 2017), African elephants (*Loxodonta africana*; Rossman et al. 2020) and parrots (*Melopsittacus undulatus*; Gallup et al. 2015; Miller et al. 2012). In contrast, evidence in some species remains equivocal. In domestic dogs (*Canis lupus familiaris*), for example, findings are mixed: while several studies report yawning contagion in response to human yawns (Joly-Mascheroni et al. 2008; Madsen and Persson 2013; Neilands et al. 2020; Romero et al. 2013; Silva et al. 2012), others have found no such effect (Buttner and Strasser 2014; Harr et al. 2009; O’Hara and Reeve 2011). Moreover, there is currently no evidence supporting contagious yawning in dog–dog dyads (Harr et al. 2009; O’Hara and Reeve 2011), suggesting that behavioural contagion in dogs might have been shaped over the course of domestication for interspecific interactions with humans rather than conspecific partners (see Call et al. 2003, and Palagi and Cordoni 2020).

Some authors have also linked behavioural contagion to empathy, arguing that perception–action mechanisms may allow observers to match the emotional states of others, giving rise to emotional contagion, which is often considered a basic form of empathy (Palagi et al. 2009, 2020; Platek et al. 2003; Preston and de Waal 2002). From this perspective, behavioural contagion is expected to vary across individuals and social contexts, reflecting differences in empathic sensitivity. For example, individuals sharing stronger social bonds or higher familiarity are predicted to show higher levels of behavioural contagion, as empathy is assumed to be stronger in these dyads (Palagi et al. 2009; Preston and de Waal 2002). Similarly, some researchers have suggested that in some species females may show higher levels of empathy and, consequently, more pronounced behavioural contagion than males, reflecting sex differences in caregiving and parental investment (Norscia et al. 2016; see Gallup and Massen 2016). However, whether such patterns provide direct evidence for emotional contagion remains debated, as they may instead reflect attentional biases toward more familiar or socially salient group members (e.g., Gallup 2021; Massen et al. 2012; Massen and Gallup 2017). From this perspective, contagion may be more likely to occur when observing individuals with prominent social roles, such as high-ranking or socially well-integrated group members. Supporting this view, in Tibetan macaques (*Macaca thibetana*), the individuals that are more central in the social network elicit significantly higher levels of contagion than more peripheral group members (Zhang et al. 2022).

To date, several studies have investigated whether behavioural contagion is modulated by the relationship quality, which has been operationalized in terms of familiarity, kinship or strength of the dyadic bond (Silk et al. 2009). In chimpanzees, for instance, contagious yawning is more frequent in ingroup or familiar individuals, as compared to outgroup or unfamiliar ones (Campbell and de Waal 2011, 2014). Similarly, contagious yawning is modulated by relationship quality in bonobos (Demuru and Palagi 2012; Palagi et al. 2014), geladas (*Theropithecus gelada*; Palagi et al. 2009) and wolves (Romero et al. 2014). However, other studies have found no effect of relationship quality on contagious yawning (chimpanzees: Massen et al. 2012; Madsen et al. 2013; spider monkeys: Valdivieso-Cortadella et al. 2023; parrots: Gallup et al. 2015), or even reported opposite patterns (in rats, *Rattus norvegicus*: Moyaho et al. 2015). In domestic dogs, some studies found that relationship quality positively affected the likelihood of yawning contagion in response to human stimuli (Joly-Mascheroni et al. 2008; Romero et al. 2013; Silva et al. 2012), whereas others reported no effect (Neilands et al. 2020; O’Hara and Reeve 2011; Madsen and Persson 2013).

With respect to sex differences, there is currently no consistent support for the prediction that females are generally more likely than males to show contagious yawning across mammals (Massen and Gallup 2017). Although one study on wolves showed that females had shorter reaction times than males to yawns produced by close social partners (Romero et al. 2014), several other studies reported no sex biases in contagious yawning (e.g., Campbell et al. 2009; Valente et al. 2023). Given that these patterns are inconsistent across species, some researchers have suggested that they may align more closely with an attention-bias account than with the hypothesis that behavioural contagion reflects emotional contagion or empathy (Massen and Gallup 2017). In chimpanzees, for example, yawns produced by males are more contagious than those produced by females (Massen et al. 2012), whereas the opposite pattern has been reported in bonobos (Demuru and Palagi 2012), suggesting attentional biases toward the dominant sex (Massen and Gallup 2017). Similarly, female geladas show higher levels of contagious yawning than males, but only when the observed yawner is female (Palagi et al. 2009). In dogs there is no evidence for sex-based modulation of contagious yawning (Neilands et al. 2020; Romero et al. 2013).

In this study, we investigated contagious yawning in wolves and wolves admixed with dogs (hereafter, admixed wolves), the latter comprising introgressed individuals of second- or later generation backcrosses to wolves (see below). From a theoretical perspective, comparing admixed and non-admixed wolves can shed light on the selective pressures shaping behavioural contagion and, more broadly, behavioural coordination. While evidence for contagious yawning is relatively well established in primates (e.g., Massen and Gallup 2017), patterns in canids are less clear. In wolves, only a single study has documented contagious yawning (Romero et al. 2014). In this study, wolves were more likely to yawn after observing a conspecific yawning, which the authors interpreted as evidence that contagious yawning may help highly social species such as wolves synchronize behavioural and physiological states within the group, thereby promoting coordination and social cohesion within the pack. In domestic dogs, however, there is no clear evidence for dog–dog contagion (Harr et al. 2009; O’Hara and Reeve 2011), and the role of familiarity and relationship quality remains inconsistent. This raises the possibility that domestication may have shaped behavioural contagion (see Palagi and Cordoni 2020), favouring the emergence of traits that enhance attention to humans (e.g., Miklósi et al. 2003) and sensitivity to human social cues (e.g., Hare and Tomasello 2005). If these traits are inherited from dogs through introgressive hybridization, admixed wolves may show contagion patterns that are intermediate between wolves and domestic dogs. Determining whether introgressed dog alleles influence behavioural contagion, therefore, may help clarify whether and how hybridization affects group coordination and social cohesion in admixed wolves, with important implications for their ecology and social behaviour.

Here, we made the following hypotheses and predictions. Given that wolves form cohesive packs characterised by strong affiliative bonds and high levels of cooperation (Packard 2003, 2019), and that contagious yawning has been documented in wolves (Romero et al. 2014) but not in dog–dog dyads (Harr et al. 2009; O’Hara and Reeve 2011), we expected that both admixed and non-admixed wolves would exhibit behavioural contagion (Prediction 1a), but that this effect would be stronger in wolves (Prediction 1b). Furthermore, if the distribution of behavioural contagion within groups is shaped by attention- and/or empathy-based biases, we predicted that contagious yawning would be more likely between individuals having stronger social bonds (Prediction 2a); when the initial yawner was socially more prominent, by having a higher rank (Prediction 2b); or when the initial yawner occupied a more central position in the social network (Prediction 2c). However, such biases may be more pronounced in cohesive groups, where individuals form stronger affiliative relationships, dominance hierarchies are more stable, and individuals may differentially allocate attention to socially salient partners (see Amici et al. 2024, for a preliminary comparison of social cohesion in admixed and non-admixed wolves). Therefore, we also predicted that the modulating effects of social bond strength, rank and centrality would be stronger in non-admixed than in admixed wolves (Prediction 2d).

## Methods

*Ethics*. Our study was purely observational and conducted in accordance with the national regulations of the countries where it took place. All the experimental procedures were approved by the Lower Saxony State Office for Consumer Protection and Food Safety (LAVES: Niedersächsisches Landesamt für Verbraucherschutz und Lebensmittelsicherheit) in Germany. Moreover, the procedures were approved by the research coordinators at Osnabrück Zoo and WildtierPark Edersee (hereafter WPE) in Kellerwald, both in Germany, and by the directors of Riserva Naturale Regionale Lago di Penne (RNRLP), Parco Faunistico del Monte Amiata (PFMA) in Arcidosso and Centro di Recupero Animanatura Wild Sanctuary (CRAWS) in Semproniano, all in Italy. Risk assessments were carried out collaboratively with the keepers of each study group, with approval granted only if the procedures were deemed to pose no risk to the animals. As all facilities allowed visitors and our observations followed standard visitor practices (i.e., observing the animals without any experimental intervention), the study was considered to pose no adverse effects to the animals.

*Study subjects*. We studied two groups of non-admixed wolves (N = 20) and three groups of admixed wolves (N = 13). All individuals in the admixed groups exhibited various combinations of anomalous morphological traits (e.g., dark or pale coats, dewclaws, depigmented nails; Ciucci et al. 2003, Galaverni et al. 2017). Genetic analysis using biparental and/or uniparental markers confirmed they were introgressed individuals from second- or later-generation backcrosses to wolves (Caniglia et al. 2020). At the time the observations, all individuals had reached sexual maturity and were considered adults (see Table 1, for the sex and age composition of the five study groups). All admixed wolves had been removed from the wild for conservation management and relocated to captivity, following implementation of management responses aimed at preventing hybridization in wild wolf populations (Bocci et al. 2015). All the study subjects lived in captive conditions, in relatively large enclosures with their conspecifics. In all groups, animals were exposed to visitors and keepers, who usually fed them every one to three days by placing dead chickens, rabbits and other potential prey in their enclosures.

**Table 1:** Location, sex, age (in years), rank and network centrality (as a measure of social integration) of the captive admixed and non-admixed wolves observed in this study. Rank and centrality values range from 0 to 1, reflecting the outcomes of dyadic agonistic interactions and an individual’s integration within the social network (see text). A value of 0 corresponds to low-ranking, poorly integrated individuals, whereas a value of 1 corresponds to high-ranking, highly integrated individuals. Dog-admixed wolves included second- or later-generation backcrosses to wolves, as determined by biparental and uniparental genetic markers (Caniglia et al. 2020)

Group	Location	Subject	Sex	Age	Rank	Centrality
Non-admixed wolves	Osnabruck	Angel	Female	5	0.000	0.627
		Ano	Male	5	0.033	0.713
		Eren	Male	7	0.224	1.000
		Grey	Female	4	0.015	0.982
		Jackal	Female	4	0.311	0.805
		Kaz	Male	7	0.488	0.850
		Lagerta	Male	5	0.549	0.737
		Noname	Female	5	0.83	0.794
		One	Female	5	0.205	0.682
		Ragnar	Male	4	1.000	0.920
		Snow	Male	7	0.123	0.882
		Witcher	Female	4	0.138	0.410
	Kellerwald	Amik	Male	5	0.000	0.219
		Anouk	Male	5	0.207	0.727
		Bagira	Male	4	0.188	0.020
		Cara	Female	2	0.213	0.431
		Charly	Male	2	0.787	0.656
		Cowalski	Male	2	0.515	0.637
		Noki	Female	8	0.816	1.000
		Nuk	Male	9	1.000	0.845
Dog-admixed wolves	Penne	Baloo	Male	7	0.048	0.625
		Balto	Male	7	0.657	1.000
		Licaone	Male	7	0.000	0.390
		Lucio	Male	7	0.454	0.812
		Neo	Male	7	1.000	0.825
		Syd	Male	7	0.554	0.707
	Semproniano	Bo	Male	11	0.481	1.000
		Nero	Male	11	1.000	0.965
		Vincent	Male	11	0.000	0.978
	Arcidosso	Koda	Female	≥ 8	0.204	0.684
		Luna	Female	10	0.988	0.949
		Ora	Female	≥ 8	1.000	1.000
		Rossa	Female	10	0.000	0.000

The Osnabrück group included 12 Hudson wolves (*Canis lupus hudsonicus*): three males from the same litter who were born in the Amsterdam Zoo (Netherlands), and nine individuals (i.e., six females and tree males) from two different litters that were born in the Osnabrück Zoo. Three of the six males had been vasectomized at the time of the study (i.e., testes were intact and gonadal endocrine function was expected to be maintained). Their 0.45 ha enclosure included open woods and a walkway along the edges, which allowed a clear view on the group. The WPE group included 8 European wolves (*Canis lupus lupus*): a breeding couple and part of their offspring from three previous litters. The 1.04 ha enclosure included grassland and shrubs, and a steep forested hillside, where wolves were not always visible. All the individuals in the group were still intact at the time of the study.

The RNRLP group included 6 male admixed wolves, all siblings, captured as pups at about 1 month of age in 2017 in Gran Sasso–Monti della Laga National Park from a dog-admixed wolf pack. They were introgressed at nuclear loci and/or at the K-locus and all shared a dog Y-haplotype (Caniglia et al. 2020), most likely representing second- or later generation backcrosses to wolves. They were initially raised in kennel boxes but relocated, at 2.5 months of age, to the RNRLP enclosure, which measured approximately 0.50 ha and included a pond and a grove. All were vasectomized at six months of age and therefore were hormonally intact. The group could be observed from an elevated visitor area that provided visual access to most of the enclosure. The CRAWS group included 3 male admixed wolves, all siblings from a litter of 10 that who was captured in May 2014 near Siena, when the pups had less than one week of age (Zingaro et al. 2014). Similar to the RNRLP group, they were introgressed at nuclear loci and/or at the K-locus, and all shared a dog Y-haplotype (Caniglia et al. 2020), most likely representing third- or later generation backcrosses to wolves. After capture, they were initially hand raised and bottle-fed by caregivers for about one month, and six (three males and three females) were then transferred to the CRAWS, where the three males had always remained together. All three males were neutered through orchiectomy during their first year of life. Their enclosure measured around 0.30 ha and included a grass clearing, a hill and, beyond the hill, another green area with trees that was not visible to the observer, who conducted the observations in front of the clearing. The PFMA group included 4 admixed wolves, all females, who were captured in different occasions in Tuscany (G. Romeo, Tuscany Region, pers. comm.). Two sisters, along with a third that died prior to the onset of this study, were from the wild admixed litter captured in 2014 near Siena and hosted in the CRAWS, from where they were transferred at 8 years of age. Two additional females were live captured as adults in 2012, one near Firenze and one near Grosseto, and were housed in separate enclosures until joining the two previous sisters in 2016 and 2022. In addition to anomalous morphological traits, all females were introgressed at the nuclear loci, likely representing third- or later generation backcrosses to wolves (Caniglia et al. 2020). All females had been sterilized, the two sisters via ovariohysterectomy, and the other two individuals via tubal ligation (Amici et al. 2024). The PFMA enclosure measured approximately 9.00 ha and included a grass clearing atop a hill surrounded by woods. Observations were conducted from a visitor turret, which provided sight of only a portion of the enclosure.

*Data collection and coding.* We conducted behavioural observations in Osnabrück from July to December 2023, at the WPE in Kellerwald from January to May 2024, at the RNRLP in Penne from April to August 2024, at the PFPMA in Arcidosso from September to October 2024, and at the CRAWS in Semproniano from May to September 2025. In each group, we collected three main types of data. First, we used all-occurrence sampling to assess group hierarchy and determine individual ranks. We recorded all visible instances of aggression (i.e., bites, lunges, chases), dominance behaviours (i.e., standing over a recumbent individual in a T-position or placing a paw on it while standing for more than two seconds), and submissive behaviours (e.g., licking a partner while maintaining a hunched posture and/or a tucked tail; Frézard and Pape 2003; Packard 2003; Pifarré et al. 2012). For each interaction, we noted the identities of the winner and loser, as well as the date. Second, we conducted 15-min individual focal observations to assess dyadic bond strength and individual social prominence, based on the proportion of time spent in proximity to other group members. Each study subject was observed in 22 focal sessions distributed across different days in a pseudorandomized order and at varying times of day to minimize temporal biases.

**Fig. 1:**
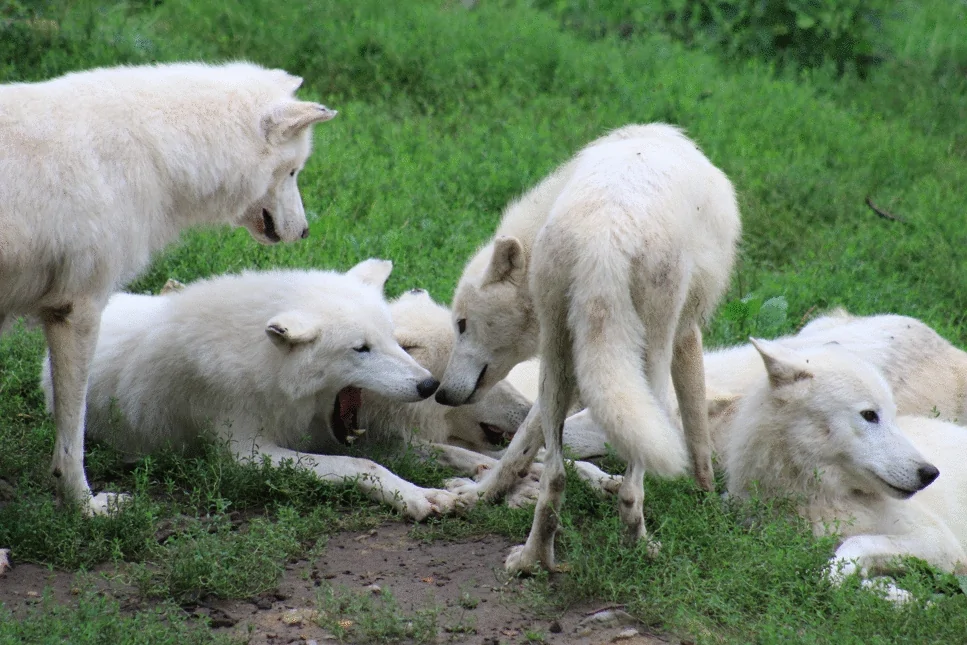
Yawn event in the non-admixed wolf group at the Osnabrück Zoo, Germany. The second wolf from the left was the yawner; the first and fourth wolves from the left were considered as having observed the yawn (i.e., their head was oriented toward the yawner during the yawn event); the third wolf from the left, in the background, was not included in the dataset, as it was not possible to understand with reasonable certainty whether the wolf had observed the yawn event or not; and the last wolf from the left was considered as not having observed the yawn

During focal sessions, we recorded the exact duration that the focal individual spent within 5 m of other group members, noting their identities. The 5 m threshold was selected based on previous observations of the study groups suggesting that this distance reliably captured affiliative social proximity between individuals while excluding more incidental spatial associations (Amici et al. 2024). Third, whenever we detected a yawning event (Fig. 1), we recorded the identities of all visible group members and coded (i) whether they observed the yawn (i.e., had their head oriented toward the yawner during the event) or not, and (ii) whether they yawned or not within the subsequent two minutes. If it was not possible to understand with reasonable certainty whether another group member had observed the yawn or not, we did not include the observation in the dataset (Fig. 1). We selected a two-minute response window based on previous findings indicating that most contagious yawning in wolves occurs shortly after the triggering event (Romero et al. 2014), and because longer observation windows would have reduced reliability when monitoring multiple individuals simultaneously. Most yawning events were recorded during focal observations, although additional events were collected ad libitum to increase sample size and robustness.

*Statistical analyses.* We conducted all analyses in R (version 4.4.2; R Core Team 2024). First, we determined the dominance hierarchy and extracted individual ranks (Table 1) using the Elo-rating method (EloRating package, version 0.43), based on all dyadic agonistic interactions with a clear winner-loser outcome Neumann and Kulik (2025). Elo scores were standardized to range from 0 to 1, with 0 corresponding to low-ranking individuals and 1 to high-ranking individuals. Second, we assessed dyadic bond strength using the Composite Sociality Index (CSI; Silk et al. 2009). CSI values were calculated by dividing the proportion of time two individuals spent in proximity by the mean proportion of time all dyads within the same group spent in proximity. CSI scores below 1 indicated lower-than-average relationship strength and values above 1 indicated higher-than-average strength. Third, we quantified individuals’ social network centrality by constructing adjacency matrices based on the time dyads spent in proximity. We used the packages asnipe (version 1.1.17; Farine 2013) and igraph (version 2.0.3; Csardi et al. 2006) to calculate individual centrality measures from these matrices (Table 1). Centrality scores ranged from 0 to 1 and reflected an individual’s level of social integration within the group: lower values indicated weaker integration, whereas higher values indicated stronger or more numerous social connections (Farine 2017).

Finally, we used generalized linear mixed models (GLMMs) to test our predictions. We constructed the dataset by entering one data point for each yawning event and each visible group member that could potentially observe it (N = 775). For each data point, we recorded: (i) the observation day (as multiple yawning events could occur on the same day); (ii) the event identity (as multiple partners could be associated with a single event); (iii) the identity of the yawner; (iv) the identity of the partner; (v) whether the partner had observed the event (0/1); and (vi) whether the partner yawned within the subsequent two minutes (0/1). We additionally included (vii) the CSI for each yawner-partner dyad, as well as the yawner’s (viii) rank and (ix) centrality as measures of social relevance within the group. Including both observers and non-observers allowed us to account for the baseline probability of yawning occurring independently of any contagion effect.

We fitted GLMMs using the *glmmTMB* package (Magnusson et al. 2021) with a binomial error distribution to model the log-odds of a partner yawning within two minutes of the initial event. As test predictors, we included three separate three-way interactions between introgression (admixed vs. non-admixed wolf, observation of the event (0/1), and (i) CSI, (ii) yawner rank and (iii) yawner centrality. As control predictors, we included yawner and partner sex (given evidence that female wolves may be more likely to exhibit behavioural contagion; Romero et al. 2014). Random intercepts included yawner identity, partner identity and event identity nested within observation day. Group identity was not included as a random effect because the number of groups was too small to reliably estimate variance components. We compared each full model to a corresponding null model that excluded the test predictors, using likelihood ratio tests (Dobson and Barnett 2008), available as R function ‘‘anova’‘*.* When the full–null comparison was significant, we used the drop1 function to assess the contribution of individual test predictors. If the three-way interaction was not significant, we refitted the model excluding that interaction and retaining the corresponding two-way interactions. We conducted post hoc pairwise comparisons for significant categorical interactions using the *emmeans* package with Tukey adjustments (Lenth 2020), with estimates back-transformed to the response scale for easier interpretability. We performed model diagnostics using the *DHARMa* (Hartig 2022) and *performance* (Lüdecke et al. 2021) packages. We detected no issues regarding residual distribution, model convergence, overdispersion or multicollinearity (maximum VIF across models = 1.68; Miles 2005).

## Results

The percentage of yawning events observed by a partner that elicited a yawn was 41% in non-admixed wolves (N = 90/221) and 24% in admixed wolves (N = 48/204). Across individuals, the proportion of yawning events that elicited a yawn after being observed was, on average (mean ± SD), 0.41 ± 0.13 in European wolves, 0.37 ± 0.25 in Hudson wolves, and 0.21 ± 0.12 in admixed wolves. When partners did not observe the yawning event, however, the percentage of yawning dropped to 6% in both non-admixed wolves (N = 10/167) and admixed wolves (N = 11/183). Across individuals, the proportion of non-observed yawning events followed by a yawn was 0.17 ± 0.15 in European wolves, 0.00 ± 0.01 in Hudson wolves, and 0.10 ± 0.13 in admixed wolves.

**Fig. 2:**
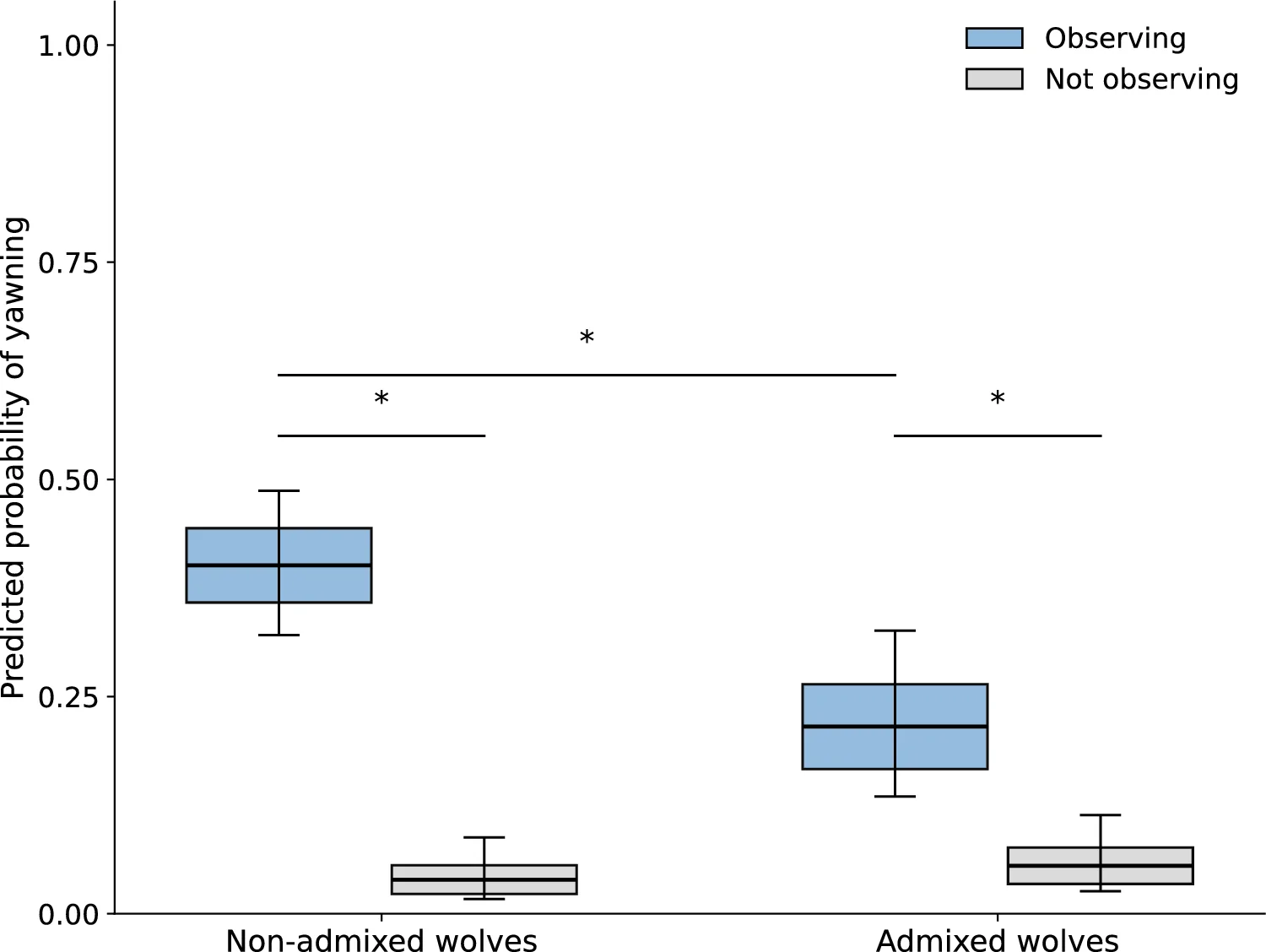
For non-admixed and dog-admixed wolves, predicted probability that other group members would yawn after observing (in blue) or not observing (in grey) another yawning event. The thick horizontal lines represent the estimated mean probabilities as assessed from the model, the horizontal ends of the boxes represent the estimated means ± SD, and the ends of the whiskers represent the 95% confidence intervals. Post-hoc significant differences are marked with an asterisk. Dog-admixed wolves included second- or later-generation backcrosses to wolves, as determined by biparental and uniparental genetic markers (Caniglia et al. 2020)

**Fig. 3:**
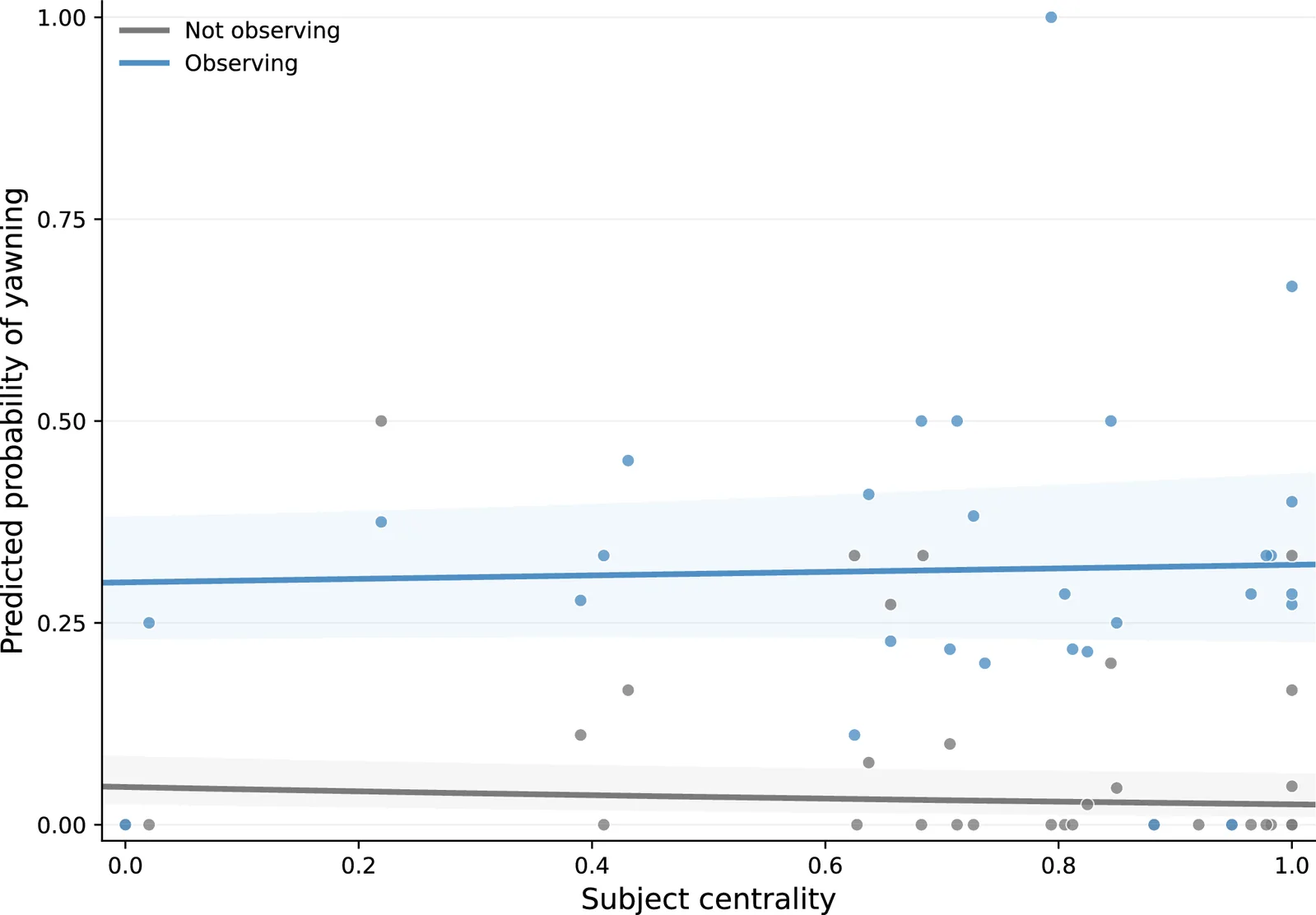
For all study animals, predicted probability that other group members would yawn after observing (in blue) or not observing (in grey) another yawning event, as a function of the yawner’s centrality in the social network. Dots represent the mean probability of yawning for each individual and the lines represent the fitted model, as described in the text

The full model significantly differed from the null one (GLMM, χ^2^ = 102.28, df = 9, *p* < 0.001), showing a significant effect of the 2-way interactions of both introgression and yawner centrality with observation of the event on the log odds of yawning (Table 2). First, post-hoc tests revealed that both admixed and non-admixed wolves were more likely to yawn after observing than not observing a yawning event (both *p* < 0.001). Moreover, the probability of a partner yawning after observing a previous yawning event was higher for non-admixed than admixed wolves (*p* = 0.016), although there was no difference between the two, if the partner had not observed the event (*p* = 0.902; Fig. 2). Second, the log odds of yawning increased with increasing yawner centrality, for both admixed and non-admixed wolves, but only after observing a previous yawning (Fig. 3).

**Table 2:** For each test predictor and control (in italics), estimates, standard deviation (SD), 95% confidence intervals (CIs), LRT, df and *p* values, with asterisks denoting significant test predictors

Predictors	Estimate	SD	95% CIs	LRT	df	P
Intercept	− 2.84	0.52	− 3.86 to − 1.83	-	-	-
Observation * Introgression	1.25	0.58	0.11 to 2.39	1	4.75	0.029*
Observation * Yawner centrality	0.74	0.32	0.12 to 1.36	1	5.38	0.020*
Observation * Yawner rank	− 0.13	0.33	− 0.77 to 0.52	1	0.15	0.702
Observation * CSI	− 0.30	0.26	− 0.81 to 0.21	1	1.26	0.262
Observation	1.55	0.38	0.79 to 2.30	-	-	-
Introgression	− 0.36	0.52	− 1.38 to 0.67	-	-	-
Yawner centrality	-0.64	0.28	− 1.19 to − 0.09	-	-	-
Yawner rank	0.11	0.30	− 0.47 to 0.69	-	-	-
CSI	0.50	0.23	0.04 to 0.95	-	-	-
*Yawner sex*	− 0.33	0.27	− 0.87 to 0.21	1	1.40	0.237
*Partner sex*	0.34	0.28	− 0.22 to 0.89	1	1.47	0.226

## Discussion

In this study, we investigated behavioural contagion in captive wolves and wolves admixed (i.e., introgressed) with dogs. Our results showed that, in both admixed and non-admixed wolves, individuals who had the opportunity to observe a yawn were significantly more likely to yawn within the subsequent two minutes than those who had not observed the event. Crucially, this effect was stronger in non-admixed than admixed wolves. Furthermore, individuals that were more socially integrated in the group were more likely to elicit behavioural contagion in both admixed and non-admixed wolves. In contrast, neither individual rank nor dyadic relationship quality between the yawner and the observer significantly influenced the probability of yawning contagion.

The presence of behavioural contagion in both admixed and non-admixed wolves supports Prediction 1a and aligns with previous findings in wolves (Romero et al. 2014). More broadly, our findings are consistent with evidence of contagious yawning across a range of social mammals and birds (e.g., Gallup et al. 2015; Miller et al. 2012; Norscia et al. 2021; Rossman et al. 2020), supporting the view that behavioural contagion represents a widespread mechanism facilitating behavioural synchrony (Duranton and Gaunet 2016; Massen and Gallup 2017). Our study extends previous work by showing that also admixed wolves, like non-admixed ones, show behavioural contagion when observing conspecifics yawning, suggesting that the perception–action mechanisms underlying this phenomenon (Chartrand and Bargh 1999; Preston and de Waal 2002; Yoon and Tennie 2010) might be preserved in admixed wolves despite introgression from domestic dogs, for which evidence of behavioural contagion in response to conspecifics is still lacking.

At the same time, we found that behavioural contagion was significantly stronger in non-admixed than in admixed wolves, supporting Prediction 1b. This difference is in line with the hypothesis that dog domestication may have favoured the emergence of social responsiveness and attentional biases toward interspecific rather than intraspecific partners, (Call et al. 2003; Miklósi et al. 2003; Hare and Tomasello 2005), possibly decreasing the likelihood of intraspecific behavioural contagion in dogs and, through the introgression of dog genes, in admixed wolves. Indeed, there is no conclusive evidence yet for dog–dog contagious yawning (Harr et al. 2009; O’Hara and Reeve 2011), although dogs have repeatedly been shown to yawn contagiously in response to human stimuli (Joly-Mascheroni et al. 2008; Romero et al. 2013; Madsen and Persson 2013; Silva et al. 2012; Neilands et al. 2020). In the future, it will be important to confirm these findings by systematically testing dog–dog social responsiveness, attentional biases and behavioural contagion in individuals living under different conditions (e.g., dogs raised by humans, dogs in kennels, free-ranging mongrel dogs), to better disentangle evolutionary and ontogenetic factors that might explain variation in these behaviours. Moreover, our findings align with previous work suggesting that social networks might be more cohesive in non-admixed than in admixed wolves (Amici et al. 2024). This indicates that the introgression of dog genes might potentially influence fine-scale mechanisms of behavioural coordination, which, in wolves, may facilitate synchrony and group cohesion (Duranton and Gaunet 2016; Conradt and Roper 2000). In the future, it will be essential to confirm the differences in behavioural coordination and group cohesion that we revealed, by including more wolf and admixed groups, possibly including recent hybrids (sensu Stronen et al. 2025) and ideally in natural settings.

Individuals’ integration in the social network mediated behavioural contagion but, in contrast to Prediction 2d, in a similar way for admixed and non-admixed wolves. In all groups, individuals that were more central in the social network were more likely to elicit yawning contagion, when the yawning event had been observed. This suggests that socially well-integrated individuals might exert greater influence on the behavioural responses of group members. Although the effect size appears modest, it mirrors findings in primates, where more central individuals elicited higher levels of contagion (Zhang et al. 2022). Social centrality may therefore represent a robust proxy for social prominence, capturing an individual’s embeddedness within the group and, consequently, its social salience (see Farine 2017). From an attention-based perspective (Massen et al. 2012; Massen and Gallup 2017), individuals that are more integrated may attract greater visual monitoring from others, thereby increasing opportunities for behavioural contagion.

In contrast, neither dominance rank nor dyadic relationship quality (CSI) significantly mediated behavioural contagion, neither in admixed nor in non-admixed wolves. The absence of a CSI effect is particularly noteworthy, as previous studies have reported higher levels of contagious yawning between closely bonded individuals in chimpanzees and bonobos (Campbell and de Waal 2011, 2014; Demuru and Palagi 2012; Palagi et al. 2014), geladas (Palagi et al. 2009) and wolves (Romero et al. 2014). Our findings therefore do not support Prediction 2a and contrast with studies linking contagion to relationship quality or familiarity. One possible explanation is methodological. Our CSI was calculated exclusively on spatial proximity (Silk et al. 2009), which may not fully capture the multidimensional nature of affiliative bonds. Wolves, in particular, are known to form complex and highly differentiated social relationships within their packs (Packard 2003, 2012). These relationships are expressed not only through spatial association but also through coordinated and cooperative activities such as hunting (MacNulty et al. 2009, 2012), breeding (Mech 1999; Packard et al. 1992) and territorial defence (Harrington and Mech 1979; Packard 2003). By relying solely on proximity as an indicator of bond strength, we may therefore have overlooked important behavioural components of social relationships, potentially limiting our ability to detect bond-related modulation of contagion. However, it is also possible that social bond strength is simply not a relevant factor in contagious yawning, in line with other literature on domestic dogs (Neilands et al. 2020; O’Hara and Reeve 2011; Madsen and Persson 2013), primates (e.g., Massen et al. 2012; Madsen et al. 2013; Pedruzzi et al. 2025; Valdivieso-Cortadella et al. 2023) and parrots (Gallup et al. 2015). Similarly, the absence of rank effects contrasts with our Prediction 2b and suggests that dominance status alone does not significantly influence behavioural contagion in admixed and non-admixed wolves.

Several limitations of our study should be acknowledged. First, the number of study groups and individuals was necessarily limited, as facilities housing groups of wolves admixed with dogs are extremely rare. Therefore, variation in enclosure size, group size and composition, as well as early life experiences, could not be fully controlled for. In particular, most admixed wolves spent few weeks in the wild with their mother and were subsequently raised in captivity, whereas the zoo-born wolves were born and raised entirely in captivity, with regular exposure to keepers and visitors. These differences were mostly graded rather than categorical, making it impossible to fully capture them with simple descriptors. It therefore cannot be excluded that such developmental differences influenced social responsiveness or attentional patterns. Moreover, wolf groups in our study were slightly larger than admixed groups, which raises the possibility that group size may have contributed to the observed difference. In principle, larger groups might promote behavioural contagion because individuals are exposed to a greater number of potential demonstrators and thus to more opportunities for contagion. In the present study, however, we accounted for this by modelling behavioural contagion as the odds that an individual yawned as a function of whether it had observed a trigger event or not. This approach reduced the likelihood that higher rates in larger groups simply reflect the presence of more potential yawners. Nevertheless, it is also possible that behavioural contagion plays a stronger functional role in larger groups, where mechanisms promoting behavioural synchrony may be especially important to maintain group cohesion and coordinated activity. If so, variation in behavioural contagion may reflect the specific socio-ecological conditions experienced by a group, rather than selective pressures working at the evolutionary level, an avenue that warrants further investigation. Second, as in most previous studies of contagious yawning (e.g., Romero et al. 2014; Massen and Gallup 2017), our data were collected in captive settings. Although our modelling approach allowed us to control for differences in the baseline occurrence of trigger events, which might also be linked to specific living conditions, caution is still warranted when extrapolating these findings to wild populations. Third, wolf-dog hybridization represents a continuum, and even if our findings were confirmed with larger samples, caution would remain necessary when generalising to more recent hybrids (sensu Stronen et al. 2025) or introgressed individuals with different degrees of admixture, as deviations from species-typical wolf behaviour may be expected to scale with the proportion of dog ancestry. Fourth, although we conducted genetic analyses to confirm introgression with dogs in our admixed groups, we did not investigate the genomic architecture potentially underlying behavioural variation. Yet recent genomic research suggests that even low levels of dog introgression may affect specific brain function and behaviour in wolves (Pilot et al. 2021), including variants involved in neurotransmission and neurodevelopment. Given the strong genetic basis of behavioural traits in dogs (Morrill et al. 2022; Salomons et al. 2021), and the extensive impact of artificial selection on the dog genome (Bergström et al., 2020; Freedman et al., 2016), it is plausible that introgressed alleles may contribute to specific behavioural variation in wild wolves subject to various extents of introgression from dogs (Leonard et al. 2013). If the observed differences between admixed and non-admixed wolves are replicated, future research integrating behavioural data with finer-scale genomic analyses will be essential to determine whether specific introgressed gene variants are associated with variation in social responsiveness and coordination. Finally, for logistic constraints, the timing of data collection varied across the five groups studied, introducing additional potential confounding factors that we could not fully account for. Temperature, for instance, is known to affect yawning behaviour (Campos and Fedigan 2009; Eldakar et al. 2015; Gallup et al. 2011; Massen et al. 2014), and although our models partially controlled for baseline yawning propensity (e.g., by also modelling individual’s likelihood to yawn when not observing the trigger event), future studies should ideally incorporate environmental temperature more explicitly as a predictor in the models. Similarly, we did not measure circulating hormone levels or account for variation in reproductive or gonadal status across individuals and groups, although this would ideally have been controlled for, given that behavioural sampling occurred both within and outside the breeding season. This may be relevant, as hormonal state might be linked to empathy-related processes and contagious yawning (e.g., Kis et al. 2020). Future work should therefore consider including hormonal status or proxies thereof to better control for its potential effects on behavioural contagion.

In conclusion, our findings support the view that dog introgression may modulate behavioural contagion in admixed wolves, possibly by modifying individual attentional biases. Given that hybridization with dogs is an emerging threat for several European wolf populations (Ciucci et al. 2026), understanding how introgression might affect behavioural processes linked to pack cohesion is of growing importance. While our findings should not be interpreted as evidence of broad behavioural disruption, they indicate that genetically detectable levels of admixture may indeed influence fine-scale social dynamics. Behavioural modifications linked to admixture could, in turn, have cascading ecological consequences: changes in attentional biases and social responsiveness may affect pack size and cohesion, social interactions within and between packs, reproductive success through alloparental care and dispersal dynamics (Newsome et al. 2017; Sparkman et al. 2012). Integrating behavioural analyses with ecological, demographic and genomic data will therefore be essential to determine whether such differences translate into measurable consequences for group coordination, cohesion and eventually the ecological role of wolves.

## Supplementary Information

Below is the link to the electronic supplementary material.

Supplementary file 1.

## Data Availability

The dataset will be published online as Supplementary Material.
